# Triad of agency, mood, and meaning: A nursing perspective on patient activation, depression, and quality of life in older adults receiving palliative oncology care

**DOI:** 10.1017/S1478951526101953

**Published:** 2026-02-27

**Authors:** Ateya Megahed Ibrahim, Donia Elsaid Fathi Zaghamir

**Affiliations:** College of Nursing, Prince Sattam Bin Abdulaziz University, Alkharj, Saudi Arabia

**Keywords:** Patient activation, depression, quality of life, palliative care, older adults, oncology, nursing

## Abstract

**Objectives:**

To examine the relationships between patient activation, depressive symptoms, and quality of life among older adults receiving palliative oncology care.

**Methods:**

A cross-sectional correlational study was conducted among 145 adults aged ≥60 years receiving palliative oncology care at King Khalid Hospital, Saudi Arabia, using stratified random sampling. Data were collected via a demographic and clinical questionnaire, the Patient Activation Measure-13 (PAM-13), the Patient Health Questionnaire-9 (PHQ-9), and the McGill Quality of Life Questionnaire–Revised (MQOL-R). Descriptive statistics, Pearson correlation, independent *t*-tests, one-way ANOVA, and multiple linear regression were performed using SPSS version 26.

**Results:**

All participants demonstrated Level 2 patient activation, with a mean PAM-13 score of 50.83 (SD = 1.04). Moderate depressive symptoms were prevalent (mean PHQ-9 = 13.56, SD = 3.48), and overall quality of life was moderate (mean MQOL-R = 55.21, SD = 10.14). Patient activation was weakly but significantly inversely correlated with depressive symptoms (*r* = −0.179, *p* < 0.05). No significant associations were found between patient activation and quality of life, or between depressive symptoms and quality of life. Regression analysis showed that patient activation, depressive symptoms, and demographics accounted for only 3.2% of the variance in quality of life (*R*^2^ = 0.032, *p* = 0.714).

**Significance of results:**

Patient activation may modestly reduce depressive symptoms but is not sufficient to improve quality of life in older adults receiving palliative oncology care. Quality of life appears influenced by broader multidimensional factors beyond activation and mood, highlighting the need for comprehensive interventions in palliative care settings.

## Introduction

In geriatric oncology, we face a painful paradox: as cancer advances and the body weakens, an older adult’s need to retain a sense of control often becomes stronger. At the bedside, this struggle is deeply human (Rodin, 2022; Schwab 2025; Zarea et al. 2025). Traditionally, *patient activation* has been defined as the willingness to adhere to treatment taking medications, keeping appointments, and following medical advice (Acquati et al. 2022; Jang and Lee 2024). Yet in palliative care, this definition feels incomplete. For these patients, activation transcends compliance; it reflects *agency* the power to shape the story of one’s remaining days. When that agency erodes, a cascade often follows: disengagement, declining mood, and eventually the loss of meaning and quality of life (QoL; Hibbard et al. 2017; Jang and Lee 2024).

Nurses witness this process most intimately. In older adults, the boundary between physical capacity and psychological well-being is delicate (Tungthongchai et al. 2023; Łuczak et al. 2024; Tang et al. 2025). The loss of physical autonomy can trigger not only sadness but a profound identity crisis that often progresses into clinical depression (Damsgaard et al. 2021; Heide 2022; Tang et al. 2025). However, such depression is frequently overlooked mistaken for the “expected” sorrow of dying rather than recognized as a treatable consequence of lost agency (Jones 2022; de Vries 2025).

This is where the “Triad” of *Agency, Mood, and Meaning* becomes crucial for nursing practice (Potter et al. 2025). Emerging evidence suggests that supporting patient activation can buffer against depression and sustain a sense of purpose, even in advanced illness (Bradley et al. 2023; Bunchuailua et al. 2025). Activation, in this context, functions as a form of existential resilience, bridging unmanaged suffering and the capacity for meaningful living (Seedsman 2021).

Despite this understanding, healthcare systems still tend to treat these dimensions separately: one plan for the cancer, another for mood, and another for QoL. This fragmented view overlooks how deeply interconnected they are (Negash and Alelign 2025; Xu et al. 2025). Withdrawal or hopelessness in older adults is too often misinterpreted as a natural part of aging or dying, rather than as a symptom of *deactivated agency* (Wheeler et al. 2023). When a nurse restores a patient’s sense of choice by optimizing comfort, enabling small acts of independence, or simply validating their voice we are not merely providing care. We are restoring humanity (Shepley 2024; Aziz 2025).

This paper explores the reciprocal links among agency, mood, and meaning in palliative oncology nursing. We argue that in older adults, patient activation functions as a vital, life-sustaining resource. By positioning these constructs within a unified framework, we propose a nursing approach that moves beyond symptom management toward empowering patients to live meaningfully until life’s final stage.

## Aim

To examine the interrelationship between patient activation (agency), mood (depression), and meaning in life, and to propose a nursing framework explaining how these constructs collectively influence QoL in older adults receiving palliative oncology care.

## Method

**Study design.** A cross-sectional, correlational study was conducted to examine the relationships among patient activation, depressive symptoms, and QoL in older adults receiving palliative oncology care. This design allows assessment of associations among the triad of Agency, Mood, and Meaning at a single point in time.

**Study setting**. The study was carried out at King Khalid Hospital and its affiliated outpatient oncology clinics in Al-Kharj, Saudi Arabia, which provide specialized palliative services for older adults with advanced cancer. These facilities offer both inpatient and outpatient care, ensuring access to a representative population.

**Study population and sample size**. The target population included older adults (≥60 years) with a confirmed cancer diagnosis receiving palliative care. The sample size was calculated using the single population proportion formula with finite population correction (FPC):

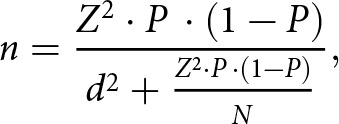


*n* = required sample size

*Z* = standard normal value (1.96) for 95% confidence level

*P* = estimated proportion (0.5 for maximum variability)

*d =* margin of error (0.05)

*N* = estimated total population

After adding a 10% non-response rate, the final sample size was 145 participants.

**Sampling technique.** Stratified random sampling was used to ensure representation across cancer types (e.g., breast, lung, gastrointestinal, and hematologic) and care settings (inpatient vs outpatient). Participants were randomly selected from the hospital registry within each stratum using computer-generated random numbers.

## Inclusion criteria

Participants were included if they

1. were aged ≥ 60 years,

2. had a confirmed cancer diagnosis,

3. were receiving palliative care services,

4. were cognitively able to provide informed consent, and

5. could communicate in Arabic or English.

## Exclusion criteria

Participants were excluded if they

1. had severe psychiatric or neurological disorders (e.g., dementia and schizophrenia),

2. were critically ill or medically unstable,

3. were unable or unwilling to provide informed consent.

## Tools of data collection

### Demographic data questionnaire

A structured demographic and clinical questionnaire, developed by the researchers based on previous literature (Polit and Beck, 2008; Hibbard et al. 2004), was used to collect participants’ basic characteristics. It included items on age, sex, marital status, educational level, living arrangement, cancer type and stage, duration since diagnosis, treatment modalities, and comorbidities. This information was used to describe the study population and examine potential associations with patient activation, depressive symptoms, and QoL.

## Patient Activation Measure-13

Patient activation was measured using the Patient Activation Measure-13 (PAM-13), developed by Hibbard and colleagues (Hibbard et al. 2005) as a shortened version of the original 22-item instrument using Rasch analysis. The PAM-13 aims to assess patients’ perceived knowledge, skills, and confidence in managing their health and healthcare, conceptualized as patient activation (Hibbard et al. 2004). The instrument consists of 13 statements reflecting a developmental continuum of activation, progressing from basic beliefs about one’s role in care to more advanced self-management behaviors (Hibbard et al. 2005). Items are rated on a four-point Likert scale ranging from strongly disagree to strongly agree, with an additional “not applicable” option. Raw scores are converted using a standardized scoring algorithm to a 0–100 scale and categorized into four activation levels, with higher scores indicating greater activation (Hibbard et al. 2005). The PAM-13 demonstrates strong construct validity supported by Rasch modeling, as well as convergent and known-groups validity through associations with health behaviors, treatment adherence, and QoL (Hibbard et al. 2004; Skolasky et al. 2011). Internal consistency reliability is consistently high, with Cronbach’s alpha values typically ≥0.80, and test–retest reliability has shown acceptable stability across diverse clinical populations, including older adults and individuals with chronic and oncological conditions (Hibbard et al. 2005).

## Patient Health Questionnaire-9

Depressive symptoms were assessed using the Patient Health Questionnaire-9 (PHQ-9), developed by Kroenke et al. (2001) as part of the Patient Health Questionnaire based on DSM diagnostic criteria for depressive disorders. The PHQ-9 is designed to measure the severity of depressive symptoms experienced over the previous 2 weeks and is widely used for both screening and severity assessment (Kroenke et al. 2001). The instrument comprises nine items corresponding to the core symptoms of major depressive disorder, including depressed mood, anhedonia, sleep disturbance, fatigue, appetite changes, concentration difficulties, psychomotor changes, feelings of worthlessness, and suicidal ideation (Kroenke et al. 2001). Each item is scored on a four-point scale from 0 (“not at all”) to 3 (“nearly every day”), producing a total score ranging from 0 to 27, with established cut-off points indicating increasing levels of depression severity (Kroenke et al. 2001). Despite including somatic symptoms that may overlap with cancer-related effects, the PHQ-9 has demonstrated strong criterion validity when compared with structured psychiatric interviews and shows good sensitivity and specificity in oncology and palliative care populations (Mitchell et al. 2016). Convergent validity has been supported through correlations with other depression measures, and internal consistency reliability is high, with Cronbach’s alpha values commonly reported between 0.80 and 0.90, alongside acceptable test–retest reliability over short intervals (Kroenke et al. 2001).

## McGill Quality of Life Questionnaire–Revised

QoL was assessed using the McGill Quality of Life Questionnaire–Revised (MQOL-R), developed by Cohen and colleagues (Cohen et al. 2019) as an updated version of the original MQOL to enhance clarity and psychometric performance while preserving its emphasis on existential aspects of QoL in life-threatening illness. The MQOL-R is specifically designed to assess QoL in individuals receiving palliative care, with particular attention to meaning, purpose, and overall well-being (Cohen et al. 2019). The instrument includes a concise set of items organized into domains assessing physical well-being, psychological well-being, existential well-being, perceived support, and overall QoL. Items are rated on numeric scales, typically ranging from 0 to 10, with higher scores indicating better perceived QoL; selected items require reverse scoring to ensure consistent directionality (Cohen et al. 2019). Domain scores are calculated as the mean of item responses, and an overall quality-of-life score can be derived according to recommended scoring procedures. The MQOL-R demonstrates strong content validity due to its development within palliative care populations and good construct validity through expected associations with symptom burden and functional status. Reliability testing has shown acceptable to high internal consistency across domains, with adequate test–retest reliability in clinically stable palliative care patients (Cohen et al. 2019).

## Ethics statement

This study was conducted in adherence to the principles of the Declaration of Helsinki. Approval for this survey was granted by the Ethics Committee of Prince Sattam bin Abdulaziz University (Approval No. SCBR-328-2024). Prior to participation, detailed information regarding the study’s objectives was provided to all nurses, along with assurances of their right to withdraw at any point.

## Statistical analysis

Data were analyzed using SPSS version 26. Descriptive statistics were calculated to summarize demographic and clinical characteristics, including frequencies, percentages, means, and standard deviations. Independent samples *t*-tests were used to examine differences in patient activation (PAM-13), depression (PHQ-9), and QoL (MQOL-R) scores between dichotomous variables (e.g., sex and comorbidities), while one-way ANOVA was applied for variables with more than two categories (e.g., age group, marital status, and education level). Pearson correlation coefficients were computed to assess relationships among PAM-13, PHQ-9, and MQOL-R scores. Finally, multiple linear regression was conducted to examine the predictive value of patient activation, depressive symptoms, and demographic characteristics on QoL (MQOL-R). Statistical significance was set at *p* < 0.05, and all tests were two-tailed.

## Results

Table 1 shows that the study sample (*N* = 145) was predominantly aged 80–89 years (42.8%), with more males (56.6%) than females. Most participants lived alone (51.7%), and educational levels were evenly distributed. Breast cancer was the most common type (30.3%), and Stage II was the most frequent cancer stage (42.8%). Table 2 indicates that all participants were at Level 2 of patient activation (mean PAM-13 = 50.83 ± 1.04), while PHQ-9 scores reveal that nearly half had moderate depression (46.9%) and 34.5% had moderately severe depression. Table 3 shows moderate QoL scores overall (MQOL-R total mean = 55.21 ± 10.14), with physical, psychological, and existential domains showing similar means (∼15), and support slightly lower (9.89 ± 4.60). Table 4 presents correlations, showing a small but significant positive relationship between patient activation and depression (*r* = 0.179, *p* < 0.05), while correlations with QoL were weak and non-significant. Table 5 demonstrates that demographic variables, including age, sex, marital status, education, living arrangement, and comorbidities, were not significantly associated with patient activation, depression, or QoL (*p* > 0.05). Finally, Table 6 shows the linear regression predicting QoL from patient activation, depression, and demographics. The model was not significant (*R*^2^ = 0.032, *F* = 0.650, *p* = 0.714), indicating that these predictors collectively explained only ∼3% of the variance in QoL. Overall, the findings suggest moderate activation and QoL with substantial depressive symptoms, but demographic and clinical factors did not significantly predict these outcomes in this sample.

**Table 1.** Demographic and clinical characteristics of participants (*N* = 145)

Variable	Category	Frequency (*n*)	Percent (%)
Age (years)	60–69	40	27.6
	70–79	43	29.7
	80–89	62	42.8
Sex	Male	82	56.6
	Female	63	43.4
Marital status	Single	31	21.4
	Married	30	20.7
	Widowed	32	22.1
	Divorced	52	35.9
Education	Illiterate	34	23.4
	Primary	40	27.6
	Secondary	35	24.1
	Higher	36	24.8
Living arrangement	Lives alone	75	51.7
	Lives with family	70	48.3
Cancer type	Breast	44	30.3
	Lung	30	20.7
	GI	38	26.2
	Hematologic	33	22.8
Cancer stage	Stage II	62	42.8
	Stage III	45	31.0
	Stage IV	38	26.2
Duration since diagnosis (months)	1–36	44	30.3
	37–72	40	27.6
	73–150	61	42.1
Comorbidities	Yes	64	44.1
	No	81	55.9

**Table 2.** Patient activation (PAM-13) and depression (PHQ-9) scores

Variable	Minimum	Maximum	Mean	SD	Categories/frequency (%)
Total PAM score	48	52	50.83	1.04	Level 2 (building confidence): 145 (100%)
PHQ-9 total score	3	20	13.56	3.48	Minimal: 1 (0.7%), mild: 20 (13.8%), moderate: 68 (46.9%), moderately severe: 50 (34.5%), severe: 6 (4.1%)

**Table 3.** Quality of life (MQOL-R) scores

Domain	Minimum	Maximum	Mean	SD
Physical	1	29	15.17	5.59
Psychological	2	28	15.08	5.60
Existential	3	28	15.08	5.59
Support	0	20	9.89	4.60
Total MQOL-R	27	79	55.21	10.14

**Table 4.** Pearson correlations between PAM-13, PHQ-9, and MQOL-R (*N* = 145)

Variable	1	2	3
1. PAM-13 total	1	0.179*	0.041
2. PHQ-9 total	0.179*	1	0.043
3. MQOL-R total	0.041	0.043	1

* Sig. (2-tailed): *p*-Value. Significance at *α* = 0.05.

**Table 5.** Relationship between demographic characteristics and patient activation, depression, and quality of life (*N* = 145)

Variable	Category	PAM-13 Mean ± SD	*t*/*F*	*p*-Value	PHQ-9 Mean ± SD	*t*/*F*	*p*-Value	MQOL-R Mean ± SD	*t*/*F*	*p*-Value
**Age group (years)**	60–69	50.20 ± 1.00	2.718	0.069	13.20 ± 3.40	0.110	0.896	54.80 ± 10.10	1.051	0.352
	70–79	50.90 ± 0.90			13.60 ± 3.50			55.00 ± 10.20		
	80–90	51.10 ± 1.20			13.80 ± 3.60			55.60 ± 10.30		
**Sex**	Male	50.79 ± 1.04	−0.229	0.819	13.56 ± 3.48	−1.439	0.152	55.21 ± 10.14	−0.619	0.537
	Female	50.87 ± 1.04			13.56 ± 3.48			55.21 ± 10.14		
**Marital status**	Single	50.55 ± 1.10	1.981	0.120	13.70 ± 3.50	0.293	0.831	55.60 ± 10.10	1.309	0.274
	Married	50.80 ± 1.00			13.55 ± 3.40			55.20 ± 10.20		
	Widowed	51.00 ± 1.10			13.50 ± 3.60			54.90 ± 10.30		
	Divorced	50.85 ± 1.00			13.60 ± 3.50			55.30 ± 10.40		
**Education level**	Illiterate	50.70 ± 1.10	0.850	0.469	13.80 ± 3.50	1.526	0.210	55.10 ± 10.30	0.266	0.849
	Primary	50.90 ± 1.00			13.50 ± 3.40			55.50 ± 10.20		
	Secondary	50.80 ± 1.10			13.60 ± 3.60			55.40 ± 10.10		
	Higher	50.85 ± 1.00			13.55 ± 3.50			55.30 ± 10.30		
**Living arrangement**	Alone	50.90 ± 1.00	0.502	0.606	13.50 ± 3.50	1.104	0.334	55.10 ± 10.20	0.528	0.591
	With family	50.80 ± 1.10			13.60 ± 3.60			55.30 ± 10.30		
**Comorbidities**	Yes	50.86 ± 1.02	−0.255	0.799	13.53 ± 3.52	0.084	0.933	53.92 ± 10.58	1.368	0.173
	No	50.79 ± 1.06			13.57 ± 3.44			55.82 ± 9.89		

Independent samples *t*-test was used for dichotomous variables.

One-way ANOVA was used for variables with more than two categories.

**Table 6.** Linear regression predicting quality of life (MQOL-R) from patient activation, depression, and demographics (*N* = 145)

Predictor	*B*	SE B	*β*	*t*	*p*
Constant	23.859	43.374	–	0.550	0.583
Total PAM-13	0.492	0.857	0.051	0.574	0.567
PHQ-9 total	0.058	0.252	0.020	0.230	0.818
Age category	1.153	1.055	0.094	1.093	0.276
Sex	1.341	1.754	0.066	0.764	0.446
Marital status	0.247	0.749	0.028	0.330	0.742
Education	0.636	0.788	0.069	0.807	0.421
Comorbidities	−2.550	1.743	−0.125	−1.463	0.146

**Model summary**: *R*^2^ = 0.032, *F*(7,137) = 0.650, *p* = 0.714. Predictors explained only ∼ 3% of the variance in QoL.

## Discussion

This study examined the interrelationships among patient activation, depressive symptoms, and QoL in older adults receiving palliative oncology care, directly addressing the core aim. The findings reveal a complex interplay of self–management capacity, mood, and existential well–being that goes beyond simple linear relationships, underscoring the need for multidimensional care strategies in palliative nursing.

Participants demonstrated moderate patient activation (all at Level 2), yet depressive symptoms were common, with nearly half reporting moderate depression and over one–third reporting moderately severe symptoms (Table 2). This pattern highlights a disconnect between perceived capacity for self–management and emotional well–being in advanced cancer. Correlation analyses showed that higher activation was weakly related to lower depressive symptoms, suggesting activation may confer some psychological buffer. This is consistent with primary care literature demonstrating inverse associations between PAM–13 and PHQ–9 scores (e.g., *r* = − 0.35) and positive associations with QoL in general populations (Magnezi et al. 2014). Additionally, older adults with multi- morbidity exhibited lower activation when depressive symptoms were higher, emphasizing the intertwined nature of mood and self–management capacity (Blakemore et al. 2016). In oncology populations, systematic reviews of PAM–13 in cancer care show that patient activation relates to engagement and treatment adherence, though evidence on its direct effect on mood varies by context (Pierre et al. 2025). This suggests that while activation may help patients feel more engaged with care, it does not fully protect against psychological distress inherent in life–limiting illnesses.

QoL scores were moderate across physical, psychological, and existential domains, with lower social support scores (Table 3). From a clinical and research perspective, this reflects the multidimensional burden of advanced cancer, where physical symptoms, social context, and existential concerns contribute substantially to well–being. Systematic reviews in developing countries affirm that social and spiritual support strongly influence QoL among palliative cancer patients, while depression and symptom intensity predict lower QoL (Haering et al. 2025). Other literature shows that higher hope, spiritual well–being, and social support correlate with better QoL and lower psychological distress, further emphasizing the importance of psychosocial and existential dimensions in palliative care (Nierop-van Baalen et al. 2020). These findings complement our data showing that moderate activation alone does not elevate QoL in this population.

The weak and non–significant correlations between activation/QoL and depression/QoL (Table 4) suggest that activation and mood may operate somewhat independently from overall QoL in advanced cancer. This aligns with studies in oncology where patient activation functions as a mediator between other factors (such as health literacy) and QoL rather than a direct predictor; higher health literacy increased activation, which in turn improved mental QoL in GI cancer patients (Haering et al. 2025). Other research indicates that while activation influences engagement in care decisions and treatment adherence, its effect on broad QoL outcomes is moderated by external factors like caregiver involvement (Acquati et al. 2022). These findings highlight that the role of activation in palliative care is context–dependent and likely intertwined with broader psychosocial and systemic influences.

No significant differences emerged for activation, depression, or QoL across age, sex, marital status, education, living arrangement, or comorbidities (Table 5). This reinforces observations from palliative and advanced cancer cohorts that clinical, existential, and psychosocial factors outweigh simple demographic predictors in shaping patient outcomes. Such results have also been documented in Saudi and international oncology studies, where depression and social support, rather than age or education per se, more strongly influence patient satisfaction and QoL (Alosaimi et al. 2022). In contrast, community and chronic disease studies often report demographic associations with activation and QoL (e.g., education and income), pointing again to the unique dynamics of advanced cancer care.

The regression model (Table 6) showed that patient activation, depressive symptoms, and demographics together explained ∼3% of the variance in QoL, with no significant individual predictors. From a research perspective, this highlights the complex, multifactorial nature of QoL in palliative oncology where variables such as symptom burden, social support, spirituality, and meaning exert stronger effects. This complexity is reflected in meta–analyses showing that early palliative care can improve psychological outcomes and QoL, particularly when interventions extend beyond symptom control to include support components (Haering et al. 2025). Other studies emphasize that existential well–being, spiritual support, and social factors are robust predictors of QoL in palliative settings factors not fully captured by activation or depression measures alone (Aslan et al. 2025).

## Implications for nursing practice and research

Collectively, these findings suggest that patient activation, while valuable, is not sufficient on its own to enhance QoL in palliative oncology. Its modest association with depressive symptoms underscores its potential as part of a holistic intervention portfolio, but the negligible direct link to QoL highlights the need to integrate activation with symptom management, psychosocial support, spiritual care, and meaning–centered care. Nursing frameworks that incorporate these domains addressing physical comfort, emotional resilience, social connectedness, and existential meaning are more likely to improve overall QoL for older adults with advanced cancer.

## Conclusion

This study contributes robust evidence that patient activation alone does not determine QoL in palliative oncology, though it may help mitigate depressive symptoms. QoL is influenced by a constellation of factors, including symptom burden, social support, existential well–being, and psychosocial contexts, advocating for comprehensive, individualized, and multidimensional nursing care.

## Recommendations

Based on the study findings, it is strongly recommended that nursing interventions in palliative oncology adopt a holistic, multidimensional approach. Strategies should integrate patient activation enhancement, psychological support, symptom management, social support facilitation, and meaning-centered or spiritual care. Structured interventions that combine self-management education with psychosocial counseling and existential support may better address the complex determinants of QoL in older adults. Additionally, routine screening for depressive symptoms using validated tools such as PHQ-9, alongside assessments of patient activation, can help identify patients at risk of emotional distress and guide tailored interventions. Incorporating caregivers and family members in care planning may further enhance support networks and overall well-being, consistent with evidence emphasizing the centrality of social and existential factors in palliative QoL outcomes.

## Study limitations

Several limitations should be considered when interpreting the findings. First, the cross-sectional design precludes causal inferences, limiting the ability to determine whether patient activation reduces depression or improves QoL over time. Second, restricted variability in patient activation (all participants at Level 2) may have reduced the ability to detect stronger relationships with QoL outcomes. Third, the study sample, though clinically diverse, was drawn from a single regional context, which may limit generalizability to other cultural or healthcare settings. Additionally, reliance on self-reported measures may introduce response or social desirability biases, particularly in sensitive domains such as mood and existential well-being. Finally, other potentially influential factors, including symptom burden, spiritual well-being, caregiver support, and socioeconomic status, were not assessed and may explain additional variance in QoL outcomes. Future longitudinal, multicenter studies incorporating broader psychosocial and clinical variables are warranted to validate and extend these findings.

## Data Availability

The original contributions presented in this study are available within the manuscript. For additional inquiries, please contact the corresponding author.

## References

[ref1] Acquati C, Hibbard JH, Miller-Sonet E, et al. (2022) Patient activation and treatment decision-making in the context of cancer: Examining the contribution of informal caregivers’ involvement. *Journal of Cancer Survivorship* 16(5), 929–939. doi: 10.1007/s11764-021-01085-9 34510365

[ref2] Alosaimi FD, Alsaleh FS, Alsughayer LY, et al. (2022) Psychosocial and clinical predictors of patient satisfaction with cancer care. *Saudi Pharmaceutical Journal: SPJ: the Official Publication of the Saudi Pharmaceutical Society* 30(4), 414–420. doi: 10.1016/j.jsps.2022.01.020 35527832 PMC9068518

[ref3] Aslan H, Çelik H and Erci B (2025) The relationship between cancer patients’ spiritual needs, quality of life and depression levels: A correlational study. *Palliative & Supportive Care* 23, e201. doi: 10.1017/s1478951525100989 41199527 PMC13166334

[ref4] Aziz F (2025) The Healing Art of Connecting with Patients. (1st ed.) Cham: Springer. doi: 10.1007/978-3-032-03624-7.

[ref5] Blakemore A, Hann M, Howells K, et al. (2016) Patient activation in older people with long-term conditions and multimorbidity: Correlates and change in a cohort study in the United Kingdom. *BMC Health Services Research* 16(1), 582. doi: 10.1186/s12913-016-1843-2 27756341 PMC5069882

[ref6] Bradley N, Dowrick C and Lloyd-Williams M (2023) Explaining how and why social support groups in hospice day services benefit palliative care patients, for whom, and in what circumstances. *Palliative Care and Social Practice* 17, 26323524231214549. doi: 10.1177/26323524231214549 38044931 PMC10693225

[ref7] Bunchuailua W, Kapol N, Krichanchai S, et al. (2025) Quality of life among thai patients with advanced cancer: Findings from the APPROACH study. *Asia‐Pacific Journal of Clinical Oncology*. doi: 10.1111/ajco.70037 PMC13007757 41131799

[ref8] Cohen SR, Sawatzky R, Russell LB, et al. (2019) Measuring the quality of life of people at the end of life: The McGill Quality of Life Questionnaire–Revised. *Palliative Medicine* 33(9), 1208–1219. doi: 10.1177/0269216319854014 27412257

[ref9] Damsgaard JB, Overgaard CL and Birkelund R (2021) Personal recovery and depression, taking existential and social aspects into account: A struggle with institutional structures, loneliness and identity. *International Journal of Social Psychiatry* 67(1), 7–14. doi: 10.1177/0020764020938812 32611264

[ref10] de Vries MFK (2025) *The Management of Loss: Humanity’s Existential Crises*. Abingdon, Oxon: Taylor & Francis.

[ref11] Haering C, Heyne S, Mehnert-Theuerkauf A, et al. (2025) The role of patient activation in mediating the effects of health literacy level on quality of life among patients with gastrointestinal cancers. *Scientific Reports* 15(1), 7295. doi: 10.1038/s41598-025-91670-0 40025154 PMC11873287

[ref12] Heide SK (2022) Autonomy, identity and health: Defining quality of life in older age. *Journal of Medical Ethics* 48(5), 353–356. doi: 10.1136/medethics-2020-107185 33741678

[ref13] Hibbard JH, Mahoney E and Sonet E (2017) Does patient activation level affect the cancer patient journey? *Patient Education and Counseling* 100(7), 1276–1279. doi: 10.1016/j.pec.2017.03.019 28330715

[ref14] Hibbard JH, Mahoney ER, Stockard J, et al. (2005) Development and testing of a short form of the patient activation measure. *Health Services Research* 40(6 Pt 1), 1918–1930. doi: 10.1111/j.1475-6773.2005.00438.x 16336556 PMC1361231

[ref15] Hibbard JH, Stockard J, Mahoney ER, et al. (2004) Development of the patient activation measure (PAM): Conceptualizing and measuring activation in patients and consumers. *Health Services Research* 39(4, Part 1), 1005–1026. doi: 10.1111/j.1475-6773.2004.00269.x 15230939 PMC1361049

[ref16] Jang SY and Lee ES (2024) Patient activation in cancer patients: Concept analysis. *Korean Journal of Adult Nursing* 36(1), 15–27. doi: 10.7475/kjan.2024.36.1.15

[ref17] Jones R (2022) *Grief on the Front Lines: Reckoning with Trauma, Grief, and Humanity in Modern Medicine*. Berkeley, California: North Atlantic Books.

[ref18] Kroenke K, Spitzer RL and Williams JBW (2001) The PHQ-9: Validity of a brief depression severity measure. *Journal of General Internal Medicine* 16(9), 606–613. doi: 10.1046/j.1525-1497.2001.016009606.x 11556941 PMC1495268

[ref19] Łuczak B, Słychan K, Bulska K, et al. (2024) Impact of physical activity on mental health in elderly population. *Quality in Sport* 21, 54087–54087. doi: 10.12775/qs.2024.21.54087

[ref20] Magnezi R, Glasser S, Shalev H, et al. (2014) Patient activation, depression and quality of life. *Patient Education and Counseling* 94(3), 432–437. doi: 10.1016/j.pec.2013.10.015 24331277

[ref21] Mitchell AJ, Yadegarfar M, Gill J, et al. (2016) Case finding and screening clinical utility of the patient health questionnaire (PHQ-9 and PHQ-2) for depression in cancer settings: A meta-analysis. *Psychological Medicine* 46(3), 531–543. doi: 10.1017/S0033291715001931 PMC4995584 27703765

[ref22] Negash BT and Alelign Y (2025) Stress and coping strategies of cancer among adult cancer patients in Hawassa University comprehensive specialized hospital cancer centre in 2024: Patient, family and health professional perspective. *BMC Cancer* 25(1), 621. doi: 10.1186/s12885-025-14023-0 40197141 PMC11974075

[ref23] Nierop-van Baalen C, Grypdonck M, van Hecke A, et al. (2020) Associated factors of hope in cancer patients during treatment: A systematic literature review. *Journal of Advanced Nursing* 76(7), 1520–1537. doi: 10.1111/jan.14344 32133663

[ref24] Pierre KJ, Verot E and Bouleftour W (2025) PAM-13 in clinical cancer care: A systematic review. *Archives of Medical Research* 56(3), 103145. doi: 10.1016/j.arcmed.2024.103145 39705861

[ref25] Polit DF and Beck CT (2008) *Nursing Research: Generating and Assessing Evidence for Nursing Practice*. Lippincott Williams & Wilkins.

[ref26] Potter PA, Perry AG, Stockert PA, et al. (2025) *Fundamentals of Nursing-E-Book: Fundamentals of Nursing-E-Book*. India: Elsevier health sciences.

[ref27] Rodin MB (2022) Maintaining functional status among older adult cancer patients and survivors. *Pathy’s Principles and Practice of Geriatric Medicine* 2, 1208–1227. doi: 10.1002/9781119484288.ch95

[ref28] Schwab K (2025) *Longevity and Retirement in the Intelligent Age: Opening New Horizons in Later Life*. Geneva, Switzerland: Schwab Academy.

[ref29] Seedsman T (2021) Mitigating the existential suffering of older people transitioning through loss and grief: Understanding the liberating influence of compassionate care. *Journal of Aging and Long-Term Care* 4(2), 19–30. doi: 10.51819/jaltc.2021.1063724

[ref30] Shepley MM (Ed.) (2024) *Peace by Design*. New York: Taylor & Francis.

[ref31] Skolasky RL, Green AF, Scharfstein D, et al. (2011) Psychometric properties of the patient activation measure among multimorbid older adults. *Health Services Research* 46(2), 457–478. doi: 10.1111/j.1475-6773.2010.01210.x 21091470 PMC3064914

[ref32] Tang L, Rasudin NSB, Dong Y, et al. (2025) Determinants of intrinsic capacity among older adults in low-and middle-income countries: A scoping review. *Belitung Nursing Journal* 11(6), 661. doi: 10.33546/bnj.4007 41312041 PMC12648234

[ref33] Tungthongchai O, Rosenberg E and Manuel Fonseca A (2023) Health promotion and physical activity in an ageing world: A reflection on a global strategies and challenges. *Revista Portuguesa de Ciências Do Desporto* (3). doi: 10.5628/rpcd.23.03.76.

[ref34] Wheeler R, Lobley M, McCann J, et al. (2023) ‘It’s a lonely old world’: Developing a multidimensional understanding of loneliness in farming. *Sociologia Ruralis* 63, 11–36. doi: 10.1111/soru.12399

[ref35] Xu J, Li Q, Gao Z, et al. (2025) Impact of cancer-related fatigue on quality of life in patients with cancer: Multiple mediating roles of psychological coherence and stigma. *BMC Cancer* 25(1), 64. doi: 10.1186/s12885-025-13468-7 39794768 PMC11721594

[ref36] Zarea K, Rafi A, Darabiyan P, et al. (2025) *Tips on Palliative Care, Hospice and Geriatric Care in Nursing*. USA: Deep Science Publishing.

